# Mechanisms of spectral and temporal integration in the mustached bat inferior colliculus

**DOI:** 10.3389/fncir.2012.00075

**Published:** 2012-10-23

**Authors:** Jeffrey James Wenstrup, Kiran Nataraj, Jason Tait Sanchez

**Affiliations:** ^1^Department of Anatomy and Neurobiology, Northeast Ohio Medical UniversityRootstown, OH, USA; ^2^Knowles Hearing Center, Roxelyn and Richard Pepper Department of Communication Sciences and Disorders, Northwestern UniversityEvanston IL, USA

**Keywords:** combination-sensitive, combination sensitivity, biosonar, echolocation, lateral lemniscus, glycinergic, medial nucleus of trapezoid body, facilitation

## Abstract

This review describes mechanisms and circuitry underlying combination-sensitive response properties in the auditory brainstem and midbrain. Combination-sensitive neurons, performing a type of auditory spectro-temporal integration, respond to specific, properly timed combinations of spectral elements in vocal signals and other acoustic stimuli. While these neurons are known to occur in the auditory forebrain of many vertebrate species, the work described here establishes their origin in the auditory brainstem and midbrain. Focusing on the mustached bat, we review several major findings: (1) Combination-sensitive responses involve facilitatory interactions, inhibitory interactions, or both when activated by distinct spectral elements in complex sounds. (2) Combination-sensitive responses are created in distinct stages: inhibition arises mainly in lateral lemniscal nuclei of the auditory brainstem, while facilitation arises in the inferior colliculus (IC) of the midbrain. (3) Spectral integration underlying combination-sensitive responses requires a low-frequency input tuned well below a neuron's characteristic frequency (ChF). Low-ChF neurons in the auditory brainstem project to high-ChF regions in brainstem or IC to create combination sensitivity. (4) At their sites of origin, both facilitatory and inhibitory combination-sensitive interactions depend on glycinergic inputs and are eliminated by glycine receptor blockade. Surprisingly, facilitatory interactions in IC depend almost exclusively on glycinergic inputs and are largely independent of glutamatergic and GABAergic inputs. (5) The medial nucleus of the trapezoid body (MNTB), the lateral lemniscal nuclei, and the IC play critical roles in creating combination-sensitive responses. We propose that these mechanisms, based on work in the mustached bat, apply to a broad range of mammals and other vertebrates that depend on temporally sensitive integration of information across the audible spectrum.

## Introduction

Our ability to perceive the location and identity of sound sources depends on information distributed across the frequency and time structure of complex acoustic signals. The peripheral auditory system performs an initial spectral analysis that separates acoustic information into a series of frequency channels. Subsequent analyses by the central auditory system combine information obtained from different frequency channels, including information about signal elements that have occurred at different times. The process of comparing information across frequency and time is termed here “spectro-temporal integration.” Spectro-temporal integration is essential for localization of sounds (Hebrank and Wright, 1974; Knudsen and Konishi, 1979; Middlebrooks, 1992; Populin and Yin, 1998), perception of conspecific vocalizations in social interactions (Park and Dooling, 1985; Boothroyd et al., 1996; Shannon et al., 2004; Moore, 2008), and analysis of sonar echoes in bats (Simmons et al., 2004; Genzel and Wiegrebe, 2008).

**Figure 1:**
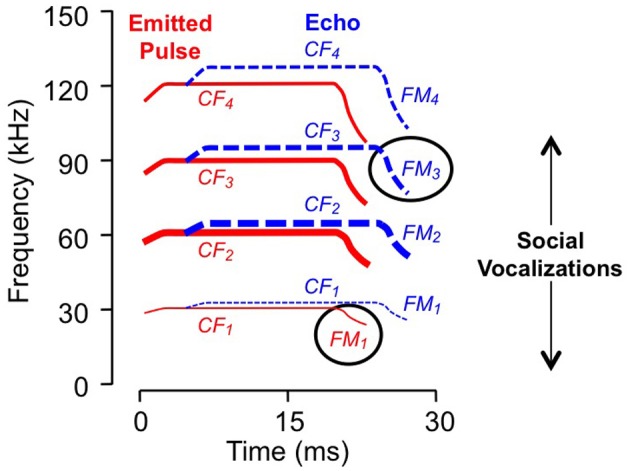
**Spectral and temporal features of vocalizations.** Schematic sonogram of echolocation signal displays emitted pulse (in red) and a Doppler (frequency) shifted and time delayed echo (in blue). Each signal is composed of CF, constant frequency; FM, frequency modulated components, with several harmonic elements (e.g., FM_1_, FM_2_, etc.). *Line thickness* indicates relative intensity: the second harmonic of the emitted pulse, near 60 kHz, is the most intense while the fundamental is usually less intense than either the second or third harmonics. *Ovals* indicate sonar elements to which the neurons in Figure 2 are tuned. Social vocalizations span the range from approximately 5–100 kHz.

This review describes mechanisms in the auditory brainstem and midbrain that contribute to spectro-temporal integration. The focus is on studies of the mustached bat (*Pteronotus parnellii*). This species displays two highly developed acoustic behaviors—echolocation and social communication—that require spectro-temporal integration for the analysis of its complex vocal signals. Spectro-temporal integration is particularly evident in the specialized responses to the mustached bat's echolocation signal (Figure 1), a complex vocalization with multiple acoustic elements. Work in the mustached bat provides an in-depth description of one form of spectro-temporal integration, *combination sensitivity*, which is characterized by neural interactions activated by distinct signal elements that occur in different frequency bands or at different times. Combination sensitivity creates selective responses to particular features of biosonar pulse-echo combinations in bats (Feng et al., 1978; Suga et al., 1978; O'Neill and Suga, 1979; Sullivan, 1982; Schuller et al., 1991; Fitzpatrick et al., 1993) and to social vocalizations in a broad range of vertebrates, including frogs (Fuzessery and Feng, 1983), birds (Margoliash and Fortune, 1992; Lewicki and Konishi, 1995), bats (Ohlemiller et al., 1996; Esser et al., 1997), and other mammals (Rauschecker et al., 1995; Kadia and Wang, 2003).

Although most studies have described combination sensitivity in the auditory forebrain, the work reviewed here reveals a sequence of spectro-temporal integrative events within the auditory brainstem and midbrain that results in the combination-sensitive neurons observed in the auditory forebrain. Along the way, these studies have identified several novel features of auditory brainstem and midbrain processing: (1) combination-sensitive response properties observed in midbrain and forebrain neurons depend on spectral convergence at distinct sites within the ascending auditory pathway, (2) spectral integration in combination sensitivity involves projections of low-frequency-tuned brainstem auditory neurons onto neurons tuned to much higher frequencies, and (3) glycinergic neurons are critically involved in both inhibitory and facilitatory combination-sensitive interactions. Further, the work identifies three regions that play key roles in creating combination sensitivity: the medial nucleus of the trapezoidal body, lateral lemniscal nuclei, and the inferior colliculus (IC). The integrative mechanisms described here are expected to apply broadly to vertebrates that utilize spectro-temporal integration to analyze complex vocal signals.

This review first considers the auditory response properties of combination-sensitive neurons and then describes mechanisms and circuitry underlying these properties.

## Combination-sensitive response properties in the inferior colliculus

**Figure 2:**
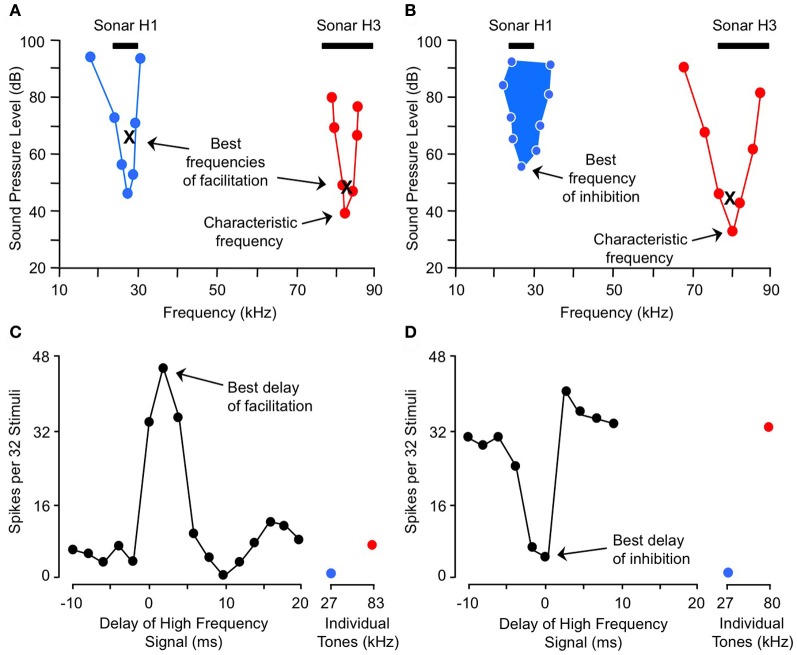
**Spectral and temporal tuning of combination sensitivity in the mustached bat's IC.** Figure shows responses of a facilitated neuron **(A** and **C)** and an inhibited neuron **(B** and **D)**. **(A)** Facilitation frequency tuning curves for high-frequency (*red*) and low-frequency (*blue*) tone bursts. These curves were obtained by fixing the frequency and level of one tone burst (X) while varying the frequency and level of a second tone burst in the other frequency band, in order to obtain threshold facilitative responses. Facilitation was defined as a response to the combination stimulus that was 20% greater than the sum of responses to the two stimuli presented separately. The high-frequency tone burst was presented at a delay corresponding to the neuron's best delay of facilitation (shown in **C**). **(B)** Excitatory (*red*) and inhibitory (*filled blue*) tuning of neuron showing combination-sensitive inhibition. The low-frequency inhibitory tuning curve was obtained by presenting a characteristic frequency tone burst at a fixed level (X), then varying the frequency and level of a low-frequency tone burst to obtain threshold inhibitory responses. Inhibition was defined as a response to the combination stimulus that was 20% less than the sum of responses to the two stimuli presented separately. The two tones were presented at the neuron's best delay of inhibition (shown in **D**). Black bars at top in **(A** and **B)** indicate frequency ranges of fundamental (H1) and third (H3) harmonic elements of biosonar call. **(C)** Delay tuning of facilitation for neuron in **(A)**. Neuron responded poorly to individual tone bursts, but strongly to the combination of facilitating tones when the high-frequency signal was delayed by 0–4 ms. Note inhibition of high-frequency response by low-frequency signal at delay of 10 ms. **(D)** Delay tuning of inhibition for neuron in **(B)**. Neuron's response to the ChF tone was inhibited by low-frequency tones when the signals were presented simultaneously. Adapted from Portfors and Wenstrup (1999), with permission.

Here we describe combination-sensitive response properties in the IC, the major nucleus of the auditory midbrain. The IC appears to be the locus of many of the integrative mechanisms underlying combination sensitivity. These mechanisms involve facilitation, inhibition, or both. Figure 2 shows spectral and temporal features of facilitatory combination sensitivity (Figures 2A,C) and inhibitory combination sensitivity (Figures 2B,D). The facilitated neuron in Figure 2A responds weakly to signals at its characteristic frequency (ChF) of 83 kHz, and is facilitated by low-frequency signals tuned to 27 kHz. The facilitatory effect of the low-frequency signal is strong only when it precedes the ChF signal by 0–4 ms, and it peaks at 2 ms (Figure 2C). The inhibited neuron in Figure 2B responds well to the ChF signal at 80 kHz, but that response is inhibited by a simultaneous signal near 27 kHz (Figures 2B,D). This inhibitory interaction is clearly distinct from the inhibition adjacent to excitatory tuning curves that is termed “sideband inhibition.” In each of these neurons, the response to the complex signal depends on distinct and well-timed spectral inputs.

In the mustached bat's IC, the majority of neurons are combination-sensitive but estimates vary substantially across studies. Between 23 and 62% of tested IC neurons display facilitation, while 24–41% of tested neurons show inhibition without facilitation (Mittmann and Wenstrup, 1995; Portfors and Wenstrup, 1999; Leroy and Wenstrup, 2000; Nataraj and Wenstrup, 2005, 2006; Macias et al., 2012). The numbers reported in these studies likely vary due to different testing methods and neuronal populations sampled, and may also differ as a result of the different sub-species of mustached bats that were studied. However, each of these studies reveals that combination sensitivity is a common response feature within the mustached bat's IC. Similar findings of combination sensitivity have not been reported in the IC of other bat species. However, studies in the big brown bat have shown that a midbrain region rostral to the IC contains combination-sensitive neurons that are tuned to pulse-echo delay (Feng et al., 1978; Dear and Suga, 1995). In the mouse IC, a smaller number of combination-sensitive neurons have been reported: approximately 16% of IC neurons are facilitatory, while 12% display inhibitory combination sensitivity without facilitation (Portfors and Felix, 2005).

In the sections below, we describe the spectral and temporal properties of these neurons in greater detail because these properties are related both to the underlying mechanisms and to the functional roles in acoustically guided behavior.

### Frequency tuning

**Figure 3:**
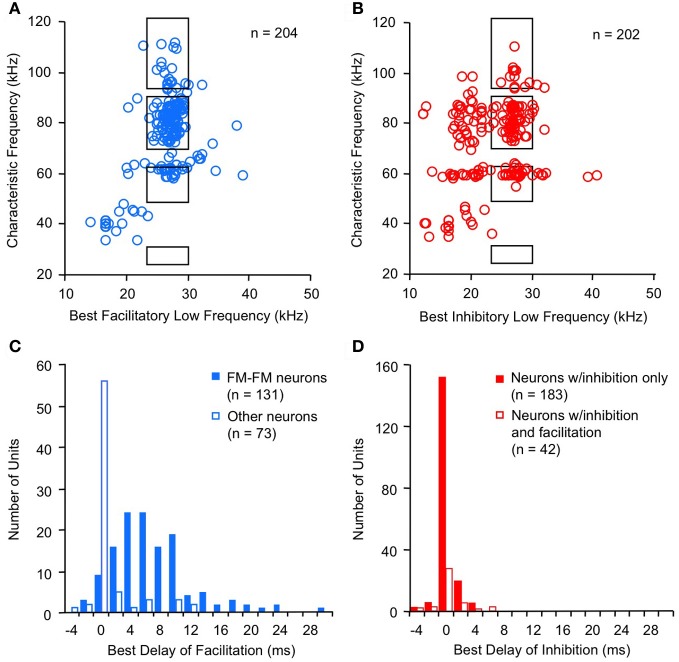
**Spectral and temporal features of combination-sensitive neurons in mustached bat IC. (A,B)** Spectral tuning of facilitation **(A)** and inhibition **(B)**. *Black rectangles* indicate frequency combinations that are present in echolocation signals. **(C,D)** Delay tuning of facilitation and inhibition. Best delays of facilitation **(C)** were broadly distributed for FM–FM neurons but tightly distributed around 0 ms delay for other types of facilitated neurons. Delay tuning of combination-sensitive inhibition **(D)** was similar for neurons showing only inhibition and for those facilitated neurons showing early inhibition. Data from Portfors and Wenstrup (1999); Leroy and Wenstrup (2000); Nataraj and Wenstrup (2005, 2006).

Note: In some of the neuroethological literature, including echolocation, the term “best frequency” is used synonymously with “ChF.” For this review we use “ChF” as it is used by many auditory neuroscientists to designate the sound frequency requiring the lowest intensity to evoke an excitatory response. We use the abbreviation “ChF” because we already use the abbreviation “CF” to designate the constant frequency (CF) component of bat sonar signals. For both low and high frequencies that evoke the strongest facilitation, we use the term “best facilitating frequency.” The best high facilitating frequency and the ChF were always very close (Portfors and Wenstrup, 1999).

Combination-sensitive neurons in the mustached bat IC are responsive to two distinct frequency bands (Figures 2A,B). These neurons typically display a clearly identifiable ChF that is almost always tuned to the higher of the two spectral bands. ChFs of these neurons range from 30 kHz to nearly 120 kHz, spanning most of the mustached bat's audible range (Figures 3A,B). Responsiveness to the lower frequency band is sometimes apparent when single tonal stimuli are presented. In many cases, however, responsiveness to the low-frequency signal is only revealed by presenting low-frequency tones in combination with the ChF tone. These tests show that most low-frequency responsiveness, whether facilitating or inhibiting, is tuned below 30 kHz (Figures 3A,B).

For the majority of combination-sensitive neurons, facilitatory or inhibitory interactions are based on frequency combinations that occur within pulse-echo sequences of the echolocation call (Figures 3A,B; Portfors and Wenstrup, 1999; Leroy and Wenstrup, 2000; Nataraj and Wenstrup, 2005, 2006). The echolocation call (Figure 1) is a brief but complex signal consisting of CF and frequency modulated (FM) elements present in multiple harmonics. The fundamental includes a relatively long (up to 30 ms) CF component near 30 kHz, terminated by a brief (<5 ms) FM down-sweep to about 23 kHz. The fundamental is attenuated by the vocal tract while the second harmonic, with CF near 60 kHz, is usually the most intense. Echoes of the emitted signal are delayed as a function of the distance between the bat and an echo source, and they are Doppler (frequency)-shifted upward as the bat approaches the echo source. CF components carry information underlying the detection and identification of sonar targets, including the fluttering insects that are the mustached bat's prey (Goldman and Henson, 1977). FM components carry information about the distance of sonar targets (Simmons, 1971, 1973; Simmons and Stein, 1980). Many combination-sensitive neurons also respond to signal elements in the mustached bat's social vocalizations. Mustached bats are highly social animals (Bateman and Vaughan, 1974) that depend on vocal signals to communicate within the dark caves that serve as roosts. The repertoire of social vocalizations spans a broad frequency range from about 5 kHz to nearly 100 kHz (Figure 1) and is considerably more varied than the stereotyped echolocation signal (Kanwal et al., 1994).

The fact that most combination-sensitive neurons are tuned to frequency combinations that occur in echolocation signals suggests that the majority of these neurons operate during echolocation behavior. Further, in all combination-sensitive IC neurons tuned to echolocation frequencies, the low-frequency facilitation or inhibition is tuned to the sonar fundamental (23–30 kHz). This indicates a special role of the fundamental in biosonar signal processing. The general view of this role is that the fundamental serves as an acoustic marker for the emitted sound: the fundamental in the emitted sound is sufficiently intense to activate auditory neurons but the fundamental in echoes is too faint to evoke a response (Suga and O'Neill, 1979; Kawasaki et al., 1988; Wenstrup and Portfors, 2011).

The frequency tuning properties of combination-sensitive neurons are indicative of their analysis of CF or FM components of biosonar echoes. Analysis of CF echoes is performed by neurons with extraordinarily sharp tuning to the CF_2_ (near 60 kHz) or CF_3_ (near 90 kHz) echo components. These neurons may be facilitated or inhibited by signals in the CF_1_ frequency range near 30 kHz or by signals in the FM_1_ range (29–23 kHz). These are designated CF–CF or FM–CF neurons, respectively. Analysis of FM echoes is performed by FM–FM neurons that are less sharply tuned to the frequencies in FM_2_, FM_3_, or FM_4_ sonar components. As described below, these differences in frequency tuning are correlated with differences in temporal properties. The neurons illustrated in Figure 2 are of the FM_1_–FM_3_ type, responding to well-timed combinations of FM_1_ and FM_3_ elements of sonar signals (Figure 1).

While the emphasis here has been on neurons tuned to biosonar frequency combinations, it is apparent from Figures 3A and B that many combination-sensitive responses are activated by frequency combinations that do not occur in sonar signals. We have speculated that these neurons play roles in the analysis of social vocalizations (Leroy and Wenstrup, 2000). Further, given the spectral overlap between sonar and social vocalizations, many combination-sensitive neurons tuned to sonar frequencies will also be activated by the multi-harmonic signals in social vocalizations (Ohlemiller et al., 1996; Esser et al., 1997).

A striking feature of the population of inhibitory neurons in Figure 3B is the large number of neurons that display an inhibitory effect tuned to signals below 23 kHz. This inhibitory effect, initially characterized on the basis of frequency tuning, also differs from inhibition tuned above 23 kHz in the sound levels required to evoke inhibition (Nataraj and Wenstrup, 2006) and in temporal properties (see section “Temporal features of low-frequency suppression”). Moreover, inhibition below 23 kHz is commonly associated with excitatory responses to the low-frequency stimulus. There is strong evidence that this suppression is the result of cochlear mechanisms related to the low-frequency “tails” of tuning curves that generate excitatory responses at high sound levels (Marsh et al., 2006; Nataraj and Wenstrup, 2006; Gans et al., 2009; Peterson et al., 2009). While not considered to be an example of combination sensitivity, it nonetheless contributes to the integrative features of neuronal responses to complex signals with energy in this <23 kHz band. For example, there is evidence that these high-ChF neurons respond well to low-frequency social vocalizations that occur at high sound levels. Furthermore, the suppressive feature of responses in the tail of the tuning curve “occludes,” or suppresses, any response to signals near the neuron's ChF (Kiang and Moxon, 1974; Portfors et al., 2002; Sheykholeslami et al., 2004). The low-frequency excitation permits these neurons to analyze acoustic signals in multiple frequency bands.

A final point related to frequency tuning is that combination-sensitive neurons occur in the IC frequency band representations that correspond to their higher, ChF response. Thus, the neuron in Figure 2A, with ChF of 83 kHz, was located within the high-frequency (>62 kHz) part of the IC. The low-frequency signal that facilitates this ChF response is tuned to 27 kHz. In order for these combination-sensitive interactions to occur, low and high-frequency-tuned inputs must converge onto single neurons where combination-sensitive response properties are created.

### Temporal sensitivity

**Figure 4:**
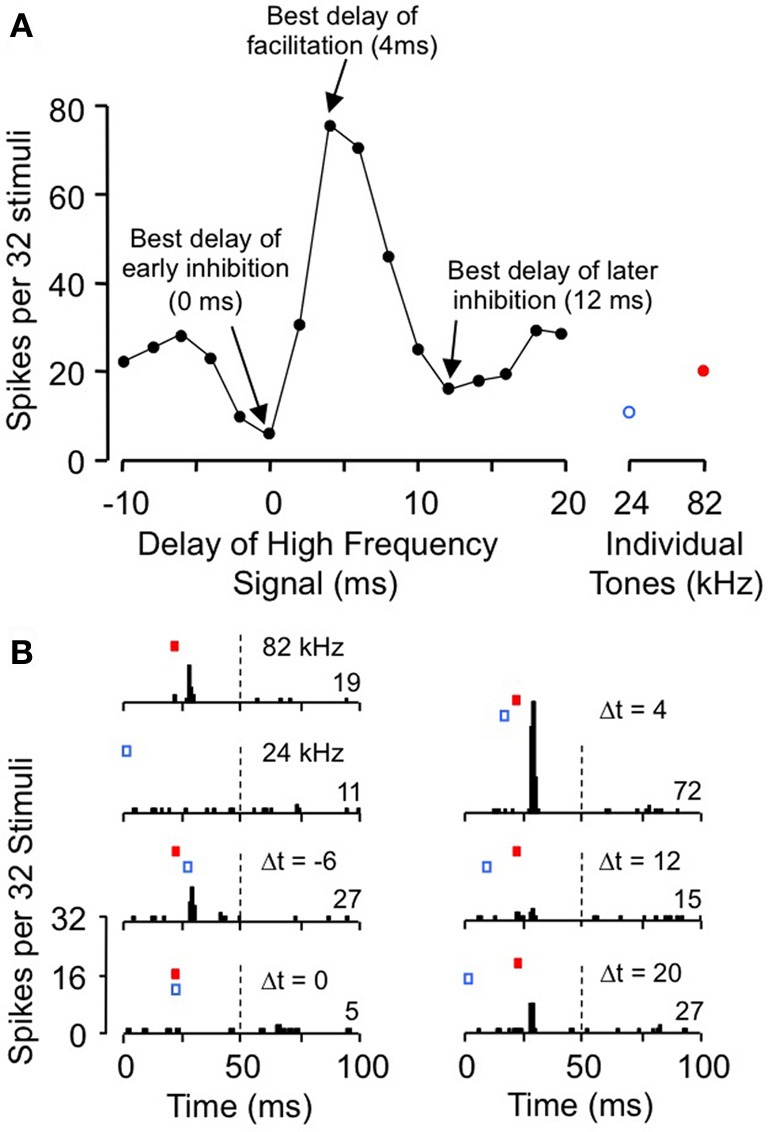
**Delay tuning of neuron showing both facilitation and inhibition. (A)** This neuron's excitatory response to ChF tones (at 82 kHz) is inhibited by low frequency tones (24 kHz) at short and long delays, but is facilitated at intermediate delays. **(B)** Post-stimulus time histograms showing temporal features of the neuron's response to tone combinations. Adapted from Nataraj and Wenstrup (2005), with permission.

The temporal features of spectral interactions in combination sensitivity are revealed in delay tests, in which the relative timing of the spectrally distinct signals is varied (Figures 2C,D). These temporal features are typically characterized by the relative timing that evokes the strongest interaction, either a peak (for facilitation) or a trough (for inhibition) in the delay function, or both (Figure 4). We refer to these as the best delay of facilitation or the best delay of inhibition. Positive delays are those for which the low-frequency signal leads the high-frequency signal. The delay functions, when combined with changes in signal duration, also reveal the duration of spectral interactions and their relationship to signal onset or offset.

#### Temporal features of facilitation

Facilitatory interactions display a broad range of best delays, ranging from −4 ms to +30 ms (Figure 3C; Portfors and Wenstrup, 1999; Nataraj and Wenstrup, 2005). A large number of neurons display the strongest facilitation when the two signals occur simultaneously (0 ms delay), with a substantial population at positive delays. Neurons with positive best delays of facilitation (in which the high-frequency signal is delayed) are likely involved in coding pulse-echo delays that occur in echolocation. Only a very few neurons show best facilitation when the high-frequency signal leads the low-frequency signal.

The distribution of best delays of facilitation differs among neurons tuned to different frequency bands. Thus, FM–FM neurons are responsible for the broad distribution of best delays. This broad range of positive delays is thought to encode sonar pulse-echo delays, in which the FM_1_ signal serves as a marker for the emitted pulse and the higher harmonic FM signal serves as a marker for the subsequent returning echo. A pulse-echo delay of 34 ms, the maximum best delay of facilitation observed in the mustached bat, corresponds to a bat-target distance of nearly 6 m. This corresponds roughly to the maximum distance of detection in many bat species (Kick, 1982; Schnitzler and Kalko, 1998; Holderied and von Helversen, 2003). The range of best facilitatory delays requires mechanisms that can delay the facilitating effect of the FM_1_ signal for up to 30 ms relative to the facilitating effect of the ChF signal.

For other combination-sensitive neurons, best delays of facilitation occur mostly when the different spectral elements are presented simultaneously (Figure 3C). This is true both for neurons that analyze the CF component of sonar signals (Portfors and Wenstrup, 1999; Nataraj and Wenstrup, 2005) as well as for neurons facilitated by frequencies that do not occur in sonar but may occur in social vocalizations (Leroy and Wenstrup, 2000; Nataraj and Wenstrup, 2005). Functionally, these neurons detect the coincidence of spectral elements in complex vocal signals. Such facilitatory interactions appear less mechanistically challenging than those in FM–FM neurons, since the facilitation only requires that the high and low-frequency excitations occur simultaneously.

**Figure 5:**
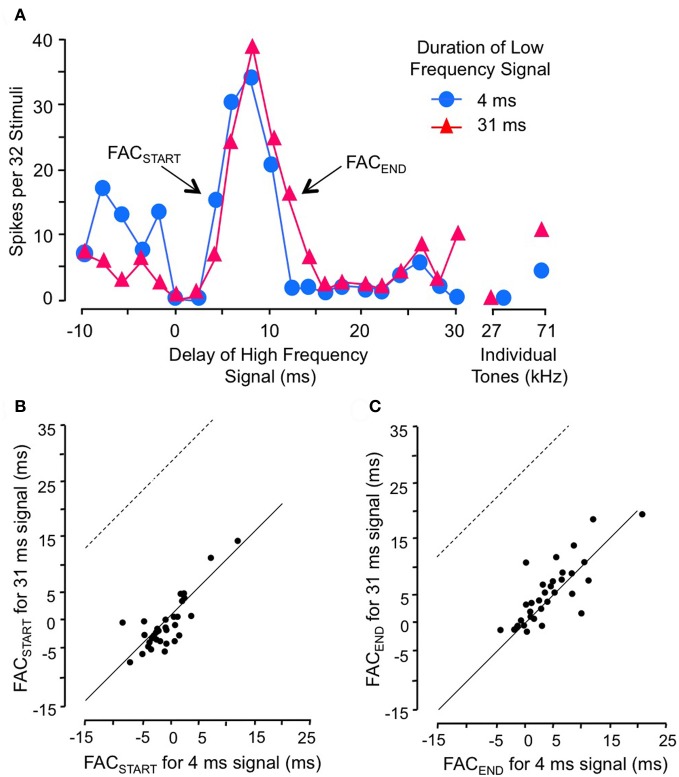
**Facilitation in combination-sensitive neurons is activated by the onset of the low-frequency signal and is phasic. (A)** Delay sensitivity of neuron was tested with two durations of low-frequency signal. **(B)** FAC_START_ in scatterplot refers to the shortest delay that evokes facilitation. Data points fall along the *solid* line, indicating that the change in low-frequency duration had no effect on this measure. Thus, facilitation is locked to the onset of the low-frequency signal. If the facilitation was locked to low-frequency offset, the delay curve is expected to shift to the right and data points in this scatter plot would fall along the *dashed* line. **(C)** FAC_END_ refers to the longest delay that evokes facilitation, a measure of the duration of the facilitating effect of the low-frequency signal. FAC_END_ is invariant with changes in low-frequency duration, indicating that the facilitating effect is phasic and independent of low-frequency signal duration. Adapted from Gans et al. (2009), with permission.

Gans and co-workers (2009) investigated how the timing of facilitation was related to the duration of the low-frequency facilitating signal (Figure 5). They found that changes in low-frequency duration had no significant effect on delay tuning (Figure 5A). The rising phase of the facilitation peak (FAC_START_) was unaffected by low-frequency duration (Figures 5A,B), indicating that the facilitation is locked to the onset of the low-frequency signal rather than to its offset. Further, the falling phase of the delay curve (FAC_END_) was unrelated to changes in signal duration, indicating that the facilitation has a fixed duration unrelated to the low-frequency signal duration (Figure 5C). Although the duration of the facilitating effect varied for different neurons, it was on average 5.3 ms. These results suggest that the onset of the low frequency signal activates a brief facilitating influence that generally cannot be extended by a longer stimulus. An input neuron with a phasic temporal pattern is consistent with these observations. Macias et al. (2012) showed that most IC facilitated neurons have delay tuning that is relatively invariant with level of the high-frequency signal. This suggests that inputs to delay-tuned neurons have level-invariant response latencies.

#### Temporal features of inhibition

For inhibitory combination-sensitive interactions, a low-frequency signal inhibits the excitatory response to the higher ChF signal (O'Neill, 1985; Mittmann and Wenstrup, 1995). Unlike facilitatory interactions, the strongest inhibition usually occurs when the two signals are presented simultaneously, i.e., having a best inhibitory delay of 0 ms (Figure 3D, Mittmann and Wenstrup, 1995; Portfors and Wenstrup, 1999; Leroy and Wenstrup, 2000; Nataraj and Wenstrup, 2006). No differences occur in the timing of inhibition for neurons responsive to different FM or CF sonar components or to signals that do not occur in sonar. In almost all cases, the inhibition is activated by the onset of the low-frequency signal (Gans et al., 2009). In general, the inhibition is phasic, lasting under 10 ms, but a smaller percentage of neurons show an inhibitory effect that could last as long as the stimulus. This suggests that the low-frequency inputs to neurons that create combination-sensitive inhibition are predominantly phasic. Neurons in the intermediate nucleus of the lateral lemniscus (INLL), which also show combination-sensitive inhibition, display similar temporal features of inhibition (Peterson et al., 2009). However, the width of inhibitory delay tuning curves may be larger among INLL neurons.

The majority of neurons (~75%) that display facilitatory combination sensitivity also show inhibitory combination-sensitive interactions (Nataraj and Wenstrup, 2005). As indicated in Figure 4, these interactions may occur at delays shorter than or longer than the delays that evoke facilitation. “Early inhibition,” generally occurring when the high and low-frequency signals are presented simultaneously, was most common, observed in 59% of facilitated neurons. The features of this early inhibition are indistinguishable from combination-sensitive neurons showing only inhibition (Figure 3D); there is no difference in the distribution of inhibitory delays, the strength of inhibition, or the width of inhibitory delay functions (Nataraj and Wenstrup, 2005, 2006). In general, early inhibition occurred in most facilitated neurons with best delays of 6 ms or longer, while early inhibition rarely occurred in neurons with best delays of facilitation of 4 ms or less. It was thus observed almost exclusively in FM–FM neurons, since these have the longest best delays of facilitation. “Late inhibition” (Figures 2C, 4) occurred in 37% of facilitated neurons. Its properties are distinct from those of early inhibition and mechanistic studies suggest a different origin (Nataraj and Wenstrup, 2005).

In general terms, inhibitory combination-sensitive interactions suppress neural responses when two spectral elements have approximately simultaneous onsets. For neurons tuned to sonar frequencies, the FM_1_ or CF_1_ inhibition suppresses the response to emitted sonar pulse. However, these neurons may respond well to echoes because the intensity of echo FM_1_ or CF_1_ components are too weak to activate the inhibition. In sonar, these neurons function as “echo-only” neurons (Mittmann and Wenstrup, 1995). For neurons that also show delay-tuned facilitation, the early inhibition suppresses response during pulse emission while the facilitation creates a strong response over a narrow range of distances. Later inhibition enhances the contrast between the strong response at facilitated delays and the responses at other delays (Portfors and Wenstrup, 1999; Nataraj and Wenstrup, 2005).

Another function of inhibitory (and facilitatory) combinatorial interactions is in analyzing spectral content in social vocalizations. In several mustached bat social vocalizations, the energy in the frequency range below 30 kHz is variable (Kanwal et al., 1994). Neurons tuned to higher frequencies, but with low-frequency combination sensitivity, may encode the level of low-frequency formants in social vocalizations (Leroy and Wenstrup, 2000). Evidence for this appears in responses of neurons with inhibitory combination sensitivity in the IC of mustached bats (Portfors, 2004) and in the auditory cortex of monkeys (Rauschecker et al., 1995).

#### Temporal features of low-frequency suppression

Suppression activated by the lowest frequencies in the mustached bat audible range, below 23 kHz, has temporal features distinct from combination-sensitive inhibition described in the preceding paragraphs. For example, the <23 kHz suppressive interactions almost always extend for the duration of the suppressing signal, rather than for a brief period following signal onset (Gans et al., 2009). Further, when the <23 kHz signal evokes an excitatory response, this response suppresses spiking responses to the high-frequency signal. These temporal features are consistent with cochlear suppression (Sachs and Kiang, 1968; Arthur et al., 1971; Kiang and Moxon, 1974), and contrast with temporal features of inhibition tuned in the 23–30 kHz range. Thus, in neurons inhibited by 23–30 kHz, excitatory response to the 23–30 kHz signals can also occur, but such spikes add to spiking evoked by high-frequency signals rather than suppress the high-frequency response (Nataraj and Wenstrup, 2006). The low-frequency cochlear-type suppression blocks responses to signals near the neuron's ChF while permitting responses to sound frequencies within the tail of the tuning curve.

## Mechanisms underlying combination-sensitive inhibition

As discussed earlier, there are several key response features of neurons that display combination-sensitive inhibition: (1) responses to ChF signals is inhibited by signals in the low (23–30 kHz) band; (2) this inhibition is usually phasic, locked to signal onset, and best when the ChF and low-frequency tones are presented simultaneously; (3) in many IC neurons, these inhibitory interactions co-occur with facilitatory interactions tuned to the same frequency bands. What mechanisms and circuitry underlie these features?

### Combination-sensitive inhibition originates in lateral lemniscal nuclei and depends on glycinergic inhibition

Although inhibitory combination-sensitive interactions have been observed in thalamic (Olsen and Suga, 1991b; Wenstrup, 1999) combination-sensitive neurons, their prominence among neurons in the mustached bat's IC suggested an origin in the IC or auditory centers below the IC. Mittmann (1997) and Portfors and Wenstrup (2001) showed that some inhibitory combination-sensitive responses occur in the INLL. Recent work in the mustached bat shows that combination-sensitive inhibition is a common response property among NLL neurons (Peterson et al., 2009). This is particularly true for INLL, but low-frequency inhibitory responses were also observed in the multipolar part of the ventral nucleus of the lateral lemniscus (VNLLm). Since previous work suggested that lower auditory brainstem structures do not display combination-sensitive inhibition (cochlear nucleus, Marsh et al., 2006), Peterson and colleagues hypothesized that these responses arise in so-called “monaural” nuclei of the lateral lemniscus, the INLL and VNLL. Collectively, these nuclei provide the largest projection to regions of the mustached bat's IC that contain combination-sensitive responses (Wenstrup et al., 1999; Yavuzoglu et al., 2011).

**Figure 6:**
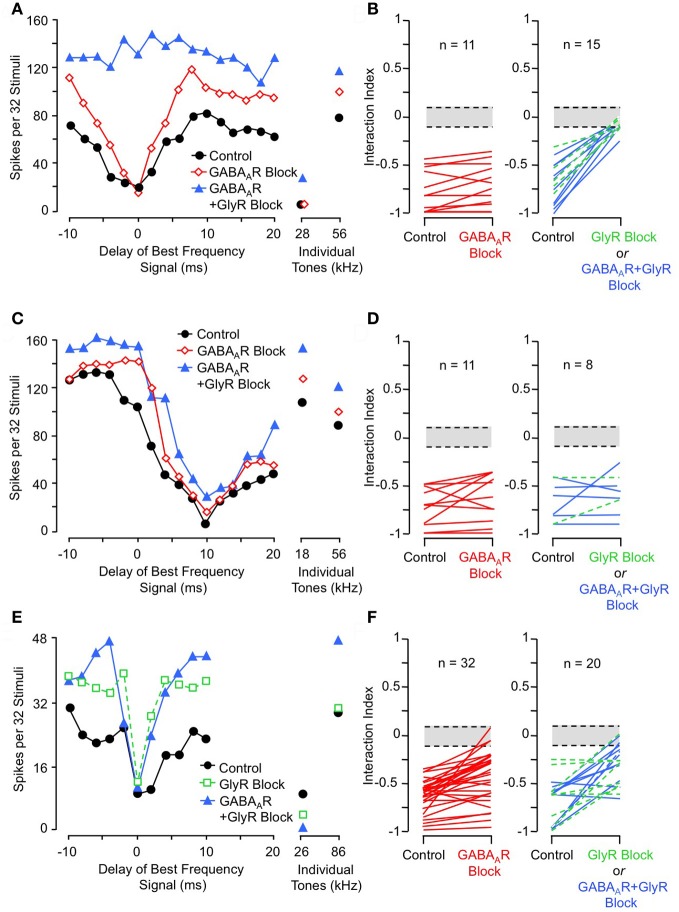
**Effects of receptor blockade on low-frequency-evoked inhibition and suppression. (A)** In INLL neuron, blockade of GABA_A_ receptor (GABA_A_R) by bicuculline did not eliminate 28 kHz inhibition, but addition of GlyR blockade by strychnine completely eliminated this inhibition. **(B)** Effects of receptor blockade on 23–30 kHz inhibition on population of tested NLL neurons. While GABA_A_R blockade alone (*at left*) did not eliminate combination-sensitive inhibition in any neuron, GlyR blockade (*at right*) always eliminated or greatly reduced inhibition evoked by 23–30 kHz signal. In **(B, D,** and **F)**, *interaction index* expresses the degree of facilitation (*positive values*) or inhibition (*negative values*). The *greyed area* indicates no significant interaction. *Green dashed lines* indicate results from GlyR receptor blockade alone, compared to *black lines* that show combined GABA_A_R and GlyR blockade. **(C)** In same INLL neuron as in **(A)**, blockade of GABA_A_R or both GABA_A_R and GlyR failed to eliminate 18 kHz suppression. **(D)** Effects of receptor blockade on <23 kHz suppression among NLL neurons. Neither GABA_A_R nor GlyR blockade eliminated suppression tuned to frequencies below 23 kHz. **(E)** In an IC neuron, blockade of GlyR did not eliminate 26 kHz inhibition, although the delay function was narrowed. Combination of GlyR and GABA_A_R blockade failed to eliminate 26 kHz inhibition. **(F)** Effects of receptor blockade on 23–30 kHz inhibition among IC neurons. Data suggest that many IC neurons inherit combination sensitivity from auditory brainstem inputs, but that some inhibitory inputs tuned to 23–30 kHz terminate onto high-ChF neurons in IC. Adapted from Peterson et al. (2009) **(A–D)** and Nataraj and Wenstrup (2005, 2006) **(E,F)**, with permission.

To test whether inhibitory interactions acting within NLL create combination-sensitive inhibition, Peterson and colleagues (2009) recorded auditory responses from NLL neurons before and after inhibitory receptor blockade. Figure 6A illustrates the effect of inhibitory receptor blockade on low-frequency inhibition in an INLL neuron. In control tests, this neuron's response to tone bursts at its ChF (56 kHz) was inhibited by 28 kHz tone bursts, and the inhibition was strongest with simultaneous presentation (0 ms delay). Blockade of GABA_A_ receptors (via bicuculline) did not reduce the low-frequency inhibition, but additional blockade of glycine receptors (via strychnine) eliminated the low-frequency inhibition. Other tests (not shown here, see Peterson et al., 2009) indicated that the elimination of inhibition is entirely attributable to GlyR blockade. Across the tested sample of lateral lemniscal neurons, low-frequency inhibition was either eliminated or greatly reduced by GlyR blockade in all neurons (Figure 6B). In contrast, GABA_A_R blockade alone was ineffective in all neurons (Figure 6B). This asymmetric effect strongly suggests that low-frequency-tone-evoked inhibition depends on a low-frequency-tuned glycinergic input to high-ChF neurons in the lateral lemniscal nuclei.

These results are striking in their contrast to similar tests conducted on suppressive responses tuned to frequencies below 23 kHz (Figures 6C,D). In the same neuron as in Figure 6A, suppression by an 18 kHz tone was unaffected by either GABA_A_R or combined GlyR and GABA_A_R blockade (Figure 6C). This was true across the entire sample of NLL neurons (Figure 6D). This result indicates that the suppressive responses to tones <23 kHz do not originate in NLL, and it is consistent with the view that this suppression is of cochlear origin.

### Combination-sensitive inhibition may be modified in IC

Studies of IC neurons support a conclusion that inhibitory combination-sensitive responses arise at levels below the IC but may be modified by interactions within the IC (Nataraj and Wenstrup, 2005, 2006). Figures 6E and F illustrate results of receptor blockade experiments. For the single neuron (Figure 6E), neither GlyR blockade nor combined GlyR and GABA_A_R blockade eliminated the 26 kHz inhibition of a high-ChF (86 kHz) response. However, note that some features of the low-frequency inhibition, especially the inhibition that occurs at delays of 2–6 ms, are reduced by GlyR blockade. Across the sample of tested neurons, inhibitory receptor blockade reduced inhibition in many neurons but eliminated it in only a few neurons (12% of tested neurons). The reduction in inhibition can occur for several reasons: it may result from blockade of low-frequency-tuned inhibitory inputs, but it may also result from the overall increase in excitation to ChF tones that occur when most inhibitory inputs are blocked.

To test whether IC neurons with high ChFs receive low-frequency inhibitory input, Peterson and colleagues (2008) used sharp electrodes to record postsynaptic potentials from combination-sensitive neurons. In the majority of neurons that showed combination-sensitive inhibition (57% of 118 neurons), they observed no low-frequency evoked inhibitory postsynaptic potentials (IPSPs) even though the high-frequency signal often evoked IPSPs. For these neurons, their inhibitory combination-sensitive response is almost certainly inherited from auditory brainstem nuclei. This is consistent with the major result of the microiontophoretic studies that inhibitory combination sensitivity in almost all IC neurons persists after local blockade of inhibitory receptors (Nataraj and Wenstrup, 2006). However, 43% of tested IC neurons show low-frequency-evoked IPSPs, indicating the presence of 23–30 kHz-tuned inhibitory inputs onto some of the high-ChF, combination-sensitive neurons. These results are consistent with the receptor blockade results showing that low-frequency inhibition is eliminated in a few neurons and is reduced in many more neurons. We believe the data support a conclusion that most IC neurons with the inhibitory combination-sensitive response property inherit that response from neurons in the lateral lemniscal nuclei, but a subset receive additional low-frequency inhibitory inputs that contribute to the IC response. In only a few IC neurons, combination-sensitive inhibition arises *de novo* through integration of high-frequency excitatory input and low-frequency inhibition.

These conclusions regarding inhibitory combination sensitivity in the IC also apply to the early inhibition observed in facilitated neurons. Nataraj and Wenstrup (2005) found that early inhibition was often reduced but rarely eliminated (8% of tested neurons) by GlyR or GABA_A_R blockade. This suggests a common mechanism or set of mechanisms underlying combination-sensitive inhibition for IC neurons, whether or not they also display facilitatory interactions.

### Circuitry underlying combination-sensitive inhibition in INLL and IC

**Figure 7:**
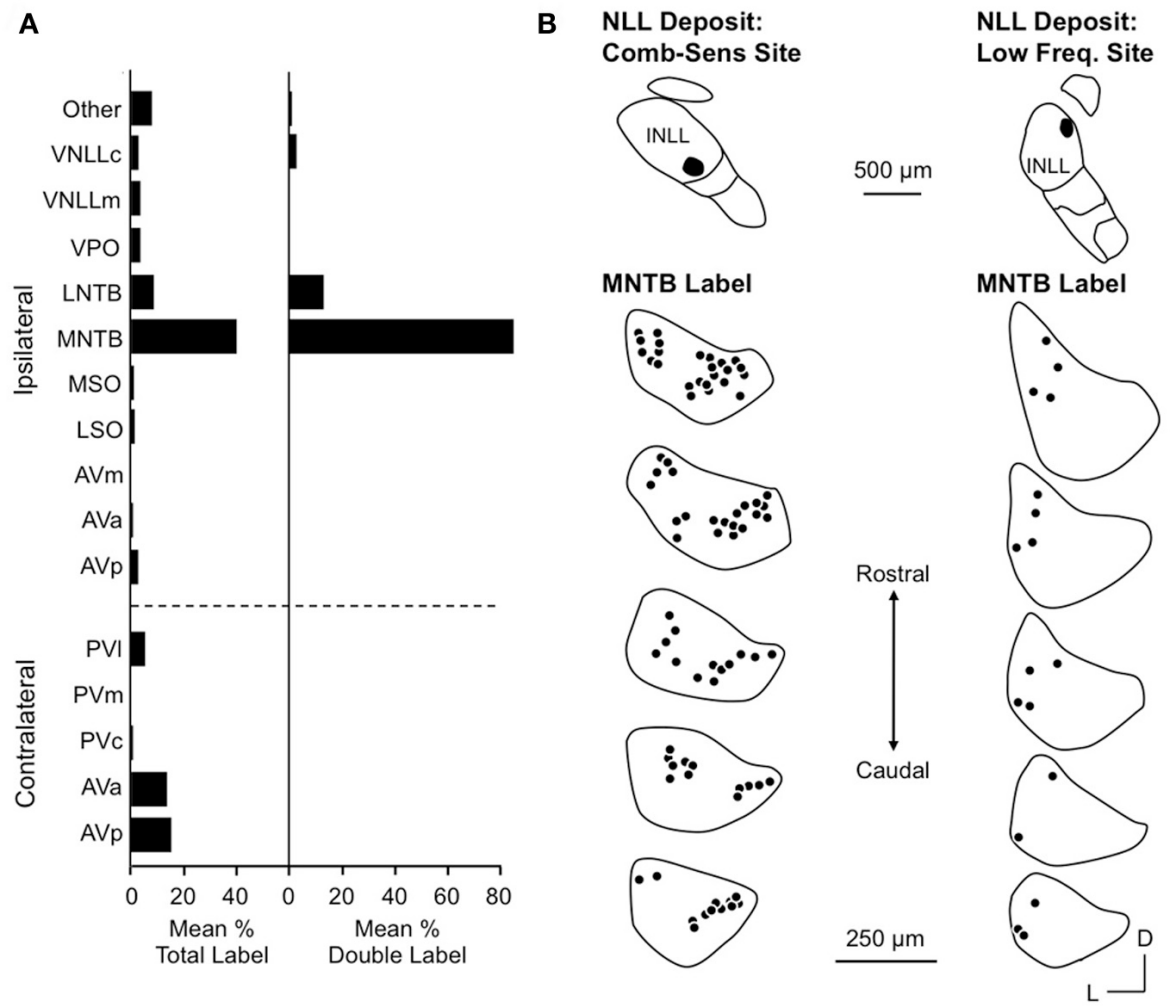
**Inputs to INLL neurons that show combination-sensitive inhibition. (A)** *Left*. Distribution of retrograde labeling after INLL deposits in five animals. *Right*. Distribution of double labeled cells (glycine-immunopositive and retrogradely labeled) after INLL tracer deposits. The ipsilateral MNTB provides the strongest glycinergic input to INLL neurons. **(B)** Comparison of retrograde label in MNTB after INLL deposits at combination-sensitive site (*left*) and low-frequency tuned site (right). MNTB labeling after combination-sensitive deposits is in both medial and lateral locations, indicating input from both low and high-frequency bands. MNTB label after low-frequency tuned deposits is located laterally, i.e., in the low-frequency representation. Adapted from Yavuzoglu et al. (2010), with permission.

Yavuzoglu et al. (2010) investigated the circuitry underlying combination-sensitive inhibition. They placed deposits of retrograde tracer at INLL recording sites featuring high ChF, inhibitory combination-sensitive response properties. The major inputs to these INLL sites are from the anteroventral cochlear nucleus (AVCN, contralateral) and the medial nucleus of the trapezoid body (MNTB, ipsilateral) (Figure 7A). Glycine immunochemistry, in combination with the retrograde transport, showed that MNTB provides the vast majority of glycinergic input (84% of input neurons) and LNTB provides most of the remainder (13%). It is noteworthy that input from VNLL, including the exclusively glycinergic columnar region, is very weak and inconsistent across experiments.

**Figure 8:**
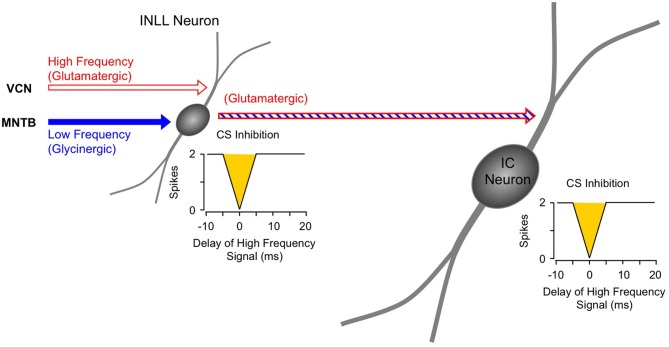
**Schematic diagram of circuitry underlying combination-sensitive inhibition in INLL and IC.** The *red and blue striped arrow* indicates sensitivity to both low and high-frequency bands.

Most inputs to combination-sensitive INLL neurons originate from regions of the cochlear nucleus or MNTB that are associated with high frequencies (Yavuzoglu et al., 2010). Thus, labeled neurons are located in the more caudal regions of AVCN and more medial part of MNTB, regions known from physiological studies and other anatomical studies to be associated with ChFs above 60 kHz (Zook and Casseday, 1985; Zook and Leake, 1989). However, Yavuzoglu and colleagues also reported retrograde labeling in the lateral part of MNTB, a region known to represent the lower frequencies in the mustached bat's audible range (Figure 7B). This consistent finding indicates that neurons in the lateral, low-frequency part of MNTB provide a spectrally unmatched input to high-frequency parts of INLL. This is the likely anatomical substrate for combination-sensitive inhibition in INLL (Figure 8).

The response properties in MNTB neurons are mostly consistent with the features of low-frequency inhibition as observed in IC combination-sensitive neurons (Gans et al., 2009). Gans and colleagues showed that low-frequency inhibition is typically but not always phasic, suggesting that low-frequency inputs to the INLL integrating neuron should be predominantly phasic. Most MNTB neurons are reported to show phasic-tonic temporal patterns that feature a tightly locked first spike and a much lower probability of subsequent spikes (Smith et al., 1998; Kopp-Scheinpflug et al., 2003; Tolnai et al., 2008). This pattern is consistent with the features of combination-sensitive inhibition in INLL neurons. Glycinergic inputs from LNTB neurons, on the other hand, do not possess the appropriate response properties, since LNTB neurons receive their primary excitation from the ipsilateral ear and project to the ipsilateral INLL. This would suggest an ipsilateral inhibitory input to INLL neurons, whereas Peterson et al. (2009) show that low-frequency inhibition is activated by the contralateral ear.

For IC neurons that display combination-sensitive inhibition, we propose that that the inhibition evoked by 23–30 kHz tones is the result of a direct excitatory projection from the inhibitory combination-sensitive neurons in INLL and perhaps VNLLm (Figure 8). The evidence described above establishes that the IC response property is, in most cases, inherited from its inputs, and that the combination-sensitive response is common in the INLL. INLL neurons project strongly to IC recording sites with combination-sensitive inhibition (Wenstrup et al., 1999; Yavuzoglu et al., 2011). Further, many and perhaps most of these INLL inputs are excitatory. This last point requires emphasis, since it is often presumed that the VNLL/INLL complex provides primarily inhibitory projections to IC. Thus, in the mustached bat, the majority of neurons in INLL are unlabeled by glycine or GABA immunocytochemistry, unlike VNLL and DNLL neurons observed in the same histological sections (Winer et al., 1995). Regions corresponding to INLL in rat and cat, sometimes considered to be the most dorsal part of VNLL, also show significant numbers of presumptive excitatory neurons (Saint Marie et al., 1997; Riquelme et al., 2001). It is less clear whether neurons in VNLLm are excitatory (Winer et al., 1995). While observations do not rule out other sources from which IC neurons inherit combination sensitivity, no other brainstem auditory nucleus is known to contain such response properties.

In a subset of IC neurons with combination-sensitive inhibition, pharmacological or physiological evidence suggests that low-frequency inhibition acts directly on high-frequency tuned neurons. Work by Yavuzoglu and colleagues (2011) provide evidence in support of this, showing that the high-frequency IC receives input from neurons in VNLL that are tuned to low frequencies and are glycinergic. Although these inputs were examined primarily in the context of facilitatory combination-sensitive interactions, the observed connections could also contribute to the inhibitory interactions observed in combination-sensitive neurons of the IC.

## Mechanisms underlying combination-sensitive facilitation

Facilitated combination-sensitive neurons detect the coincidence of excitation evoked by acoustic signals in two distinct frequency bands (Olsen and Suga, 1991a,b; Portfors and Wenstrup, 1999). Mechanistic explanations must account for several response features: (1) neuronal integration of high and low-frequency tuned responses, (2) a range of best delays of facilitation, from 0 ms (simultaneous low and high-frequency elements) to more than 30 ms lag in the high-frequency signal, (3) phasic facilitation locked to signal onset; and (4) co-occurrence of facilitatory and inhibitory interactions within single IC neurons.

### Combination-sensitive facilitation originates in IC and depends on glycinergic input

Facilitative combination-sensitive responses are abundant in several areas of the mustached bat's auditory cortex (Suga and O'Neill, 1979; Suga et al., 1983; Suga and Horikawa, 1986; Edamatsu et al., 1989; Fitzpatrick et al., 1998) and thalamus (Olsen and Suga, 1991a,b; Wenstrup and Grose, 1995; Yan and Suga, 1996a; Wenstrup, 1999). In IC, these facilitative responses are commonly observed in frequency representations above 30 kHz (Mittmann and Wenstrup, 1995; Portfors and Wenstrup, 1999; Leroy and Wenstrup, 2000). Comparative physiological recordings across auditory brainstem and midbrain nuclei suggest that combination-sensitive facilitation originates in the IC. Thus, no facilitatory responses occur in the cochlear nuclei (Marsh et al., 2006) and very few have been recorded in the lateral lemniscal nuclei (Mittmann, 1997; Portfors and Wenstrup, 2001).

**Figure 9:**
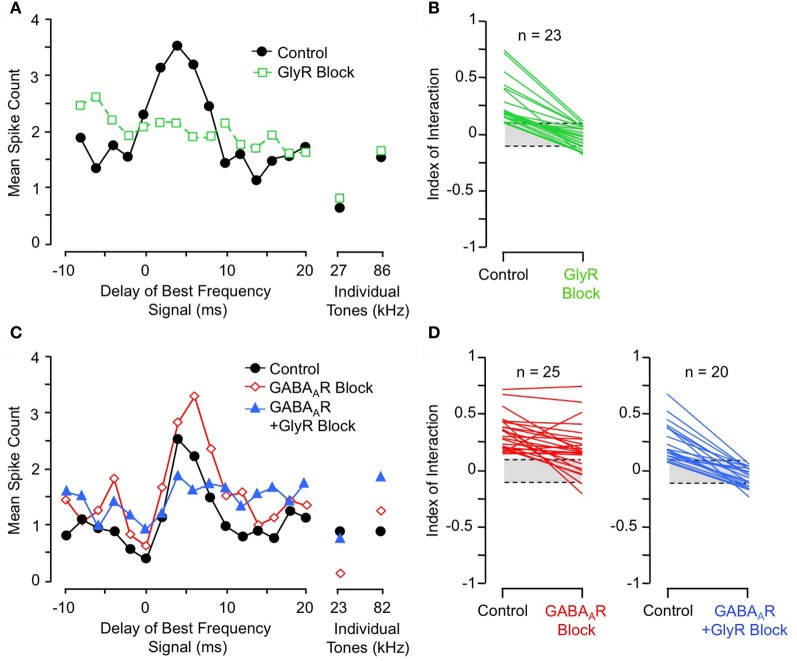
**Glycine receptor blockade eliminates combination-sensitive facilitation in IC neurons. (A)** In IC neuron, blockade of GlyRs eliminates 27 kHz facilitation of 86 kHz ChF response. **(B)** Effects of GlyR blockade on low-frequency facilitation among IC neurons. In all neurons, GlyR blockade eliminated or greatly reduced combination-sensitive facilitation. **(C)** In an IC neuron, blockade of GABA_A_Rs did not eliminate facilitation, but addition of GlyR blockade eliminated facilitation. **(D)** In most IC neurons, facilitation was not eliminated by GABA_A_R blockade, but addition of GlyR blockade always eliminated the facilitation. Adapted from Nataraj and Wenstrup (2005), with permission.

Further support comes from pharmacological studies of inhibitory receptor blockade. Several studies of IC neurons show that blockade of the glycine receptor by strychnine eliminates or greatly reduces facilitation in all combination-sensitive neurons (Wenstrup and Leroy, 2001; Nataraj and Wenstrup, 2005; Sanchez et al., 2008). Figure 9A shows an example of the general trend (Figure 9B): GlyR blockade eliminates facilitation that is strongest at 4 ms delay, even though the excitatory discharge to single tones is unaffected. In contrast, blockade of GABA_A_Rs is usually ineffective (Figures 9C,D); in all such cases, addition of GlyR blockade eliminated combination-sensitive facilitation (Figure 9D). Further studies using the GABA_A_R blocker gabazine suggest that GABA_A_Rs play no role in facilitation (Sanchez et al., 2008). The robust effect of GlyR blockade, seen across three separate studies, argues strongly that facilitation originates in the IC. It also suggests an important role for glycinergic inhibition in the facilitatory mechanism. Since GlyR blockade eliminates facilitation for both sonar and non-sonar combinations of spectral elements, and for facilitation over a broad range of best delays (Nataraj and Wenstrup, 2005; Sanchez et al., 2008), glycinergic input appears to be a fundamental contributor to mechanisms underlying combination-sensitive facilitation.

### Mechanisms of combination-sensitive facilitation: roles of excitation and inhibition

In general, facilitation in the central nervous system is thought to depend on excitatory inputs. Proposed mechanisms of facilitation in response to combinations of sensory inputs include the summation of subthreshold excitatory inputs (Finn et al., 2007), enhancement through postsynaptic glutamate receptors (Binns, 1999), and combinations of excitatory inputs with inhibitory inputs that generate post inhibitory rebound (Casseday et al., 1994). Studies in the mustached bat reveal a novel mechanism by which distinct inhibitory inputs create facilitation, presumably through dual post inhibitory rebound.

**Figure 10:**
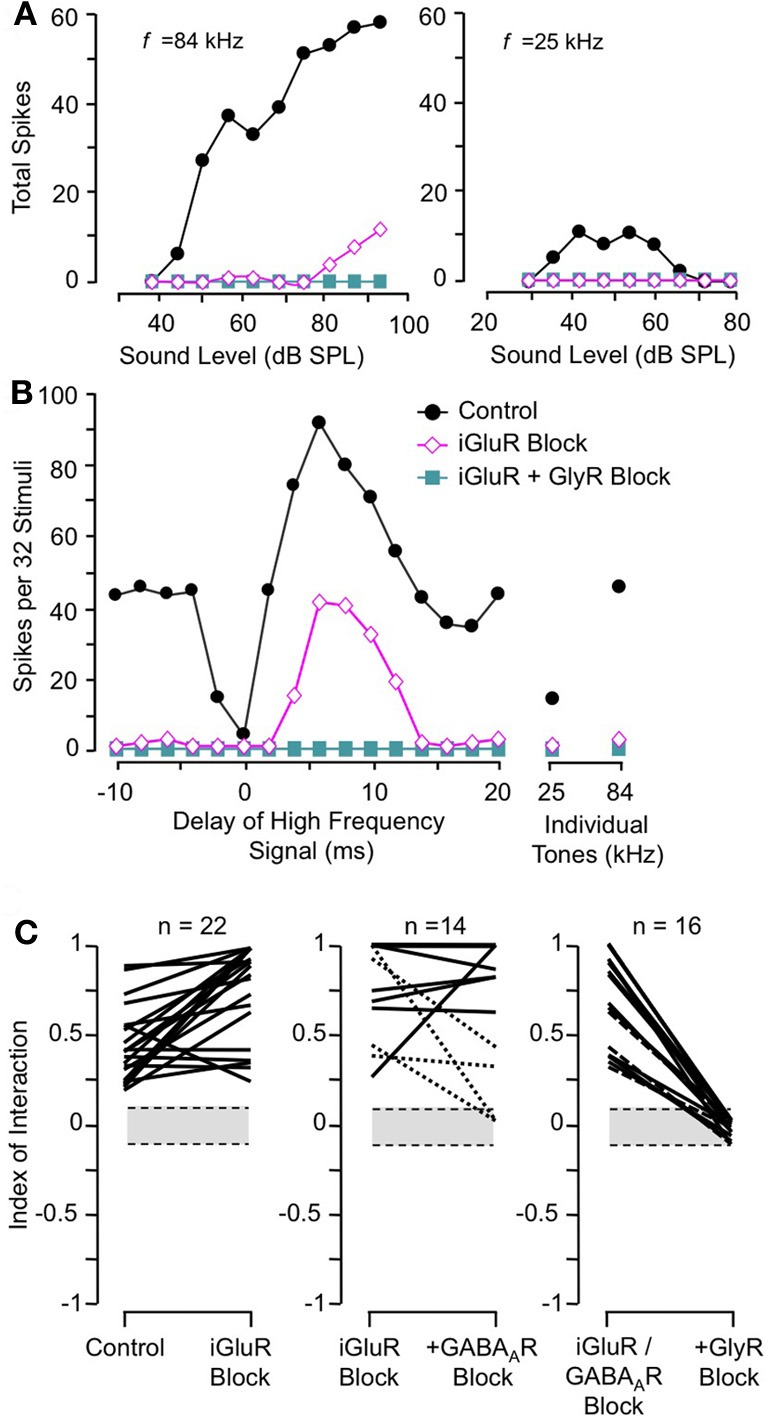
**Glutamate receptors (iGluRs) play no role in combination-sensitive facilitation. (A)** Responses to ChF and low-frequency tones are eliminated by iGluR blockade. **(B)** Blockade of iGluRs eliminated excitatory responses to single tones, but facilitated combination-sensitive responses persisted. Only the application of a GlyR blocker eliminated facilitatory interactions. **(C)** Effects of receptor blockade on low-frequency facilitation among IC neurons. In all neurons, iGluR blockade failed to eliminate facilitation, but GlyR blockade always eliminated facilitation. Blockade of GABA_A_Rs generally did not eliminate facilitation. Adapted from Sanchez et al. (2008), with permission.

Sanchez and colleagues (2008) compared the contributions of excitatory and inhibitory transmission in creating combination-sensitive facilitation in the IC. Unexpectedly, excitatory neurotransmission by glutamate played no role. They found that blockade of AMPA receptors (AMPARs) and/or NMDA receptors (NMDARs) had no effect on combination-sensitive facilitation, even though glutamate receptor blockade eliminated spike discharge in response to single tonal stimuli (Figures 10A,B). Blockade of GABA_A_ receptors in addition to glutamate receptor blockade generally failed to eliminate the facilitation or even change the number of spikes evoked by single tonal or combination stimuli. Only blockade of the glycine receptor was effective in eliminating response facilitation, and it was successful in all tested neurons. (Figure 10C).

**Figure 11:**
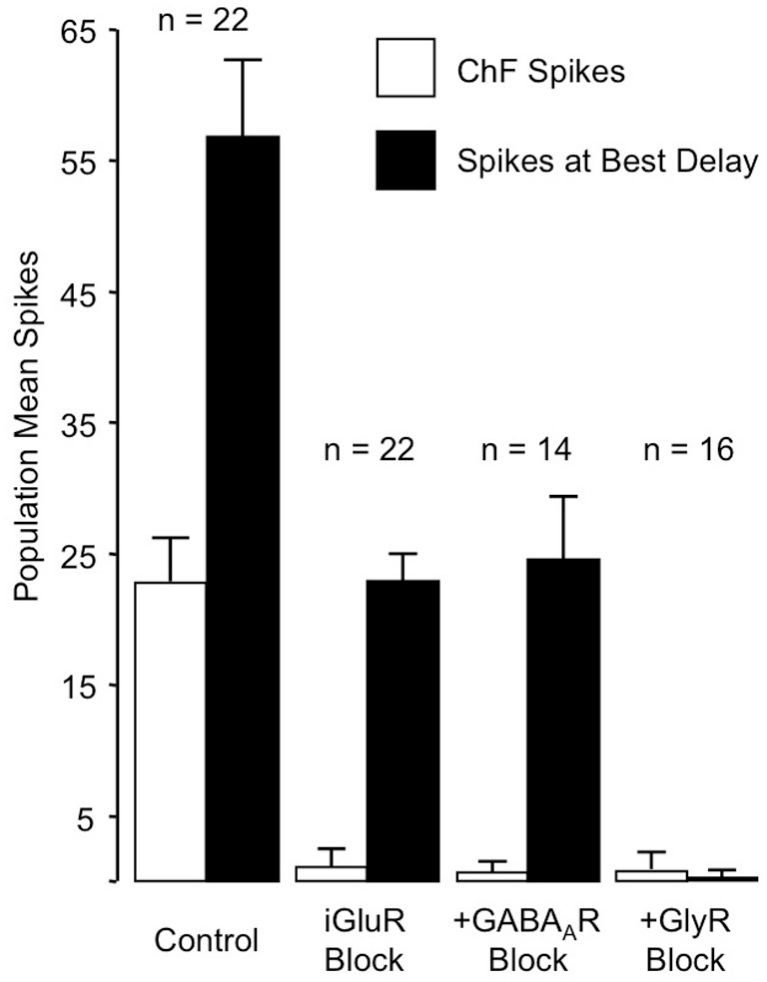
**Primary role of GlyR receptors in the facilitated response of IC neurons.** Graphs show number of spikes evoked by ChF and combination stimuli at best delay, averaged across the number of neurons in the sample. iGluR blockade eliminates spikes evoked by ChF tones, but does not eliminate facilitated spikes evoked by combination stimuli. The addition of GABA_A_R blockade to the iGluR blockade (+GABA_A_R Block) has little additional effect on the facilitated spikes. Facilitation spikes are only eliminated by addition of the GlyR blockade (+GlyR Block). Neither + GABA_A_R Block nor +GlyR Block revealed additional excitatory response to the ChF response, suggesting that iGluR blockade successfully eliminated glutamatergic excitation to the neurons. Adapted from Sanchez et al. (2008), with permission.

These results rule out any contribution of ionotropic glutamate receptors to the basic mechanism for combination-sensitive facilitation in IC. Not only were single tonal responses eliminated by glutamate receptor blockade, but application of the GABA_A_ and glycine receptor blockers did not uncover residual excitation that could result from incomplete glutamate receptor blockade (Figure 11). As a result, glycine modulation of glutamatergic transmission, as may occur in the auditory brainstem (Turecek and Trussell, 2001), or inheritance from auditory cortico-collicular inputs (Yan and Suga, 1999; Suga et al., 2000) are not viable mechanisms for combination-sensitive facilitation. Further, mechanisms that depend on a combination of glutamate excitation and glycine-evoked post-inhibitory rebound (Casseday et al., 1994; Wenstrup and Leroy, 2001) are not sufficient to account for the facilitatory interaction.

**Figure 12:**
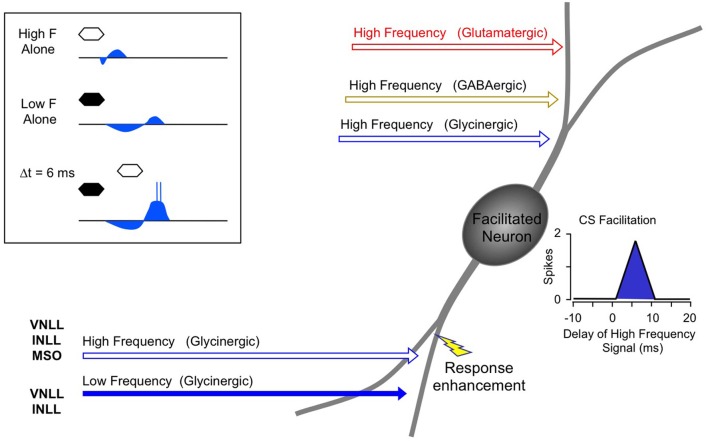
**Schematic diagram of mechanisms and circuitry underlying combination-sensitive facilitation in IC.** *Inset* shows hypothesized mechanism of post-inhibitory rebound. Neuron receives a variety of high frequency inputs tuned to its ChF (*upper right*) that do not appear to interact with glycinergic inputs related to facilitation (*lower left*). *Response enhancement* refers to hypothesized boost in the glycine rebound potentials that allows the facilitation signal to reach the spike trigger zone.

Instead, the work by Sanchez and colleagues suggests that combination sensitivity in the mustached bat's IC depends exclusively on well-timed glycinergic inputs tuned to different sound frequencies. Sanchez et al. hypothesized that the facilitatory effect of glycinergic inputs could result either from coincidence of post-inhibitory rebound excitations (Figure 12, *inset*) or direct glycine-evoked depolarizations as occurs in birds and developing mammals (Hyson et al., 1995; Kandler and Friauf, 1995; Lu and Trussell, 2001). To test these hypotheses and to further explore the mechanisms underlying combination sensitivity, Peterson and colleagues (2008) obtained intracellular recordings from facilitated combination-sensitive neurons in the mustached bat's IC. The surprising result was that in all but one tested neuron, there was no evidence of low-frequency-evoked transient hyperpolarization OR depolarization that could be related to the inputs that create response facilitation. In addition, the intracellular recordings showed no evidence of shunting inhibition that might conceal inhibitory inputs. Because the facilitatory interactions originate in IC neurons, the authors concluded that the glycinergic inputs underlying facilitation must be electrically segregated from the soma, isolated in specific dendritic regions.

The Peterson et al. study (2008) did not resolve the question of the mechanism underlying combination-sensitive facilitation. Any mechanism must also explain the delay tuning observed in many of these neurons, accounting for delays in low-frequency excitation that can exceed 30 ms. These delays are not present in the response latencies of auditory brainstem neurons that provide input to the IC (Klug et al., 2000; Portfors and Wenstrup, 2001; Marsh et al., 2006). In our view, a post-inhibitory rebound mechanism is most capable of generating the delayed excitation necessary for combination-sensitive facilitation. In such a mechanism (Figure 12; Peterson et al., 2008), glycinergic input tuned to the lower frequency signal may create an extended period of hyperpolarization with variably timed rebound. When this excitation coincides and colocalizes with high-frequency, glycine-evoked excitation, response facilitation occurs. We further propose that an additional mechanism, such as voltage-gated sodium channels placed nearer to the soma, is necessary to generate a sufficient voltage boost to allow the facilitation signal to reach the neuron's spike trigger zone. This would explain why the low-frequency input is hidden from the “view” of somatic intracellular recording, even while the facilitation signal is clearly detectable (Peterson et al., 2008).

For whichever mechanism applies, three observations strongly support a conclusion that the site of facilitation is isolated from other inputs to IC neurons. First, facilitating interactions are unaffected by glutamatergic and GABAergic inputs (Figures 10, 11; Sanchez et al., 2008). Second, glycine receptors inhibit glutamatergic responses to ChF tones while contributing to facilitation (Wenstrup and Leroy, 2001; Nataraj and Wenstrup, 2005; Sanchez et al., 2008). Third, GABA_A_- receptors inhibit glutamatergic responses to best frequency tones in the same neurons that display facilitation dependent on glycine receptors. The presence, in the same neuron, of inhibitory and facilitatory chloride-mediated influences suggests that effects of increased chloride conductance are local within the neuron. Our interpretation of these observations is that facilitatory interactions are segregated on specific dendrites, away from other sources of input (Figure 12). Regardless of where the inputs are located on IC neurons, this facilitatory response—due to differently tuned glycinergic inputs—violates the segregation of differently tuned neurons within the tonotopically organized ascending auditory pathway. How does facilitation arise in the context of the auditory system's tonotopic organization?

### Circuitry underlying combination-sensitive facilitation in IC

**Figure 13:**
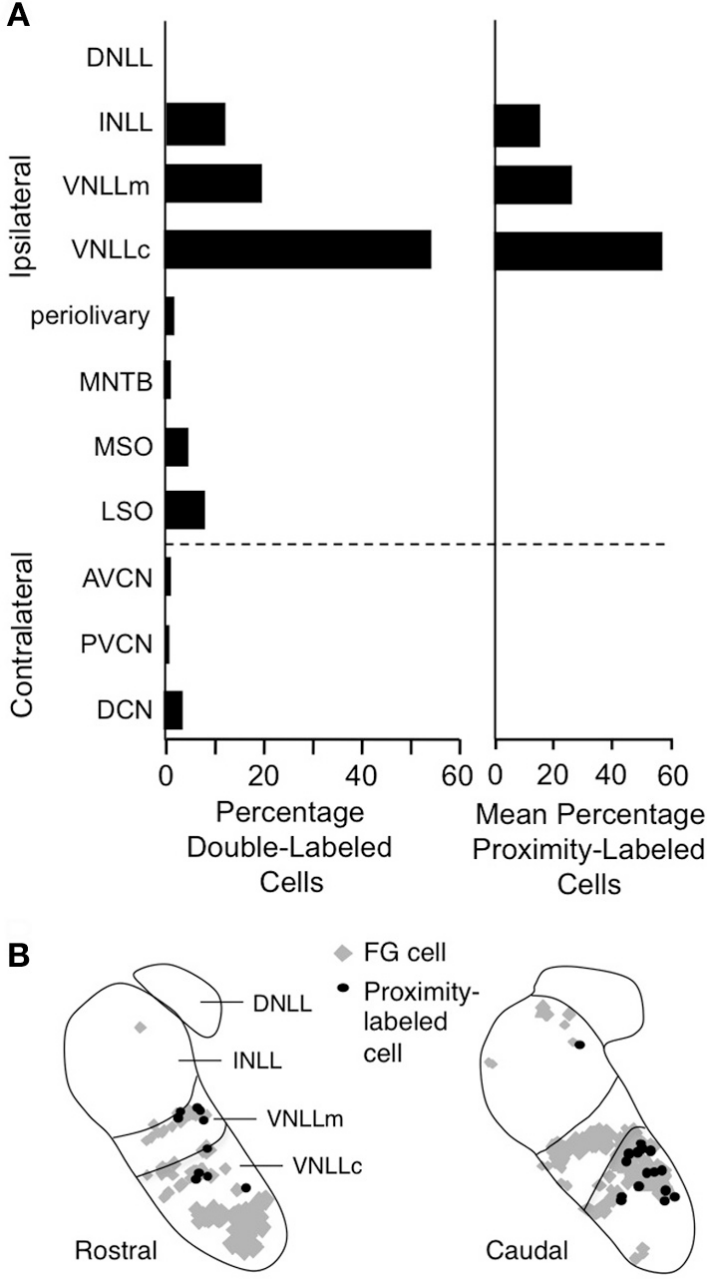
**Lateral lemniscal nuclei provide key glycinergic inputs to facilitated combination-sensitive neurons in IC. (A)** *Left*. Average percentages of neurons double-labeled by tracer (FluoroGold, FG) deposited at IC combination-sensitive recording sites and by glycine immunohistochemistry. This represents the distribution of glycinergic inputs to IC regions containing facilitated combination-sensitive neurons. *Right*. Distribution of “proximity-labeled neurons”; these neurons are retrogradely labeled by the IC tracer deposits and within 50 μm of a labeled terminal resulting from deposit of a second tracer in a low frequency part of AVCN. This represents neurons that likely receive input tuned to 23–30 kHz and project to high-ChF, combination-sensitive recording sites in IC. **(B)** Locations of these “proximity labeled” cells in VNLL and INLL from one experiment. Adapted from Yavuzoglu et al. (2011), with permission.

Facilitated combination-sensitive neurons in the mustached bat's IC receive a broad range of inputs (Wenstrup et al., 1999; Yavuzoglu et al., 2011) that activate glutamatergic, GABAergic, and glycinergic mechanisms (Wenstrup and Leroy, 2001; Nataraj and Wenstrup, 2005; Sanchez et al., 2008). Of these, only a subset of the glycinergic inputs contributes to response facilitation. Further, IC facilitated neurons must receive glycinergic inputs tuned to two frequency bands: its ChF and, in most cases, the 23–30 kHz band that contributes to most combination-sensitive facilitation. Yavuzoglu et al. (2011) combined glycine immunohistochemistry with retrograde tract tracing to identify the sources of these glycinergic inputs (Figure 13). Tracers deposited at facilitated, combination-sensitive recording sites in IC resulted in tracer-glycine double-labeled neurons in VNLL and INLL, and to a lesser extent in the lateral and medial superior olive (LSO and MSO, respectively). Together, these four auditory brainstem nuclei accounted for ~93% of glycine-immunolabeled neurons that project to facilitate combination-sensitive neurons in the IC of the mustached bat (Figure 13A). Of these inputs, the source of facilitating high-frequency glycinergic input almost certainly arises from the VNLL and INLL. Because ipsilateral LSO neurons are not excited by contralateral stimuli (Covey et al., 1991), and only a small number of MSO neurons provide glycinergic inputs to IC (Winer et al., 1995; Yavuzoglu et al., 2011), it is unlikely that LSO and MSO contribute significantly to combination-sensitive responses.

To identify the source(s) of low-frequency input, Yavuzoglu et al. (2011) placed a deposit of a retrograde tracer at an IC site displaying combination-sensitive facilitation, and a second, anterograde tracer at a 23–30 kHz recording site in the AVCN. Only VNLL and INLL contained neurons retrogradely labeled by the IC in close proximity to anterogradely labeled boutons from the low-frequency AVCN deposit (Figure 13B). These experiments confirm that VNLL and INLL are the sources of the low-frequency, glycinergic inputs that underlie combination-sensitive facilitation. Of these, it appears that the columnar part of VNLL (VNLLc), with distinctive morphology and physiological properties, is the most likely source of these low-frequency inputs (Figure 13B).

Several features of neurons in VNLLc are particularly well suited to the functional properties needed for facilitated neurons in IC. First, all of these neurons are thought to be glycinergic (Winer et al., 1995; Vater et al., 1997). Second, most of these neurons in bats have onset temporal patterns (Metzner and Radtke-Schuller, 1987; Covey and Casseday, 1991; Portfors and Wenstrup, 2001) that correspond closely to the inputs required to create the transient, onset-type facilitation observed in most IC neurons (Gans et al., 2009). Third, the level-tolerant response latencies of most VNLLc neurons (Covey and Casseday, 1991) are consistent with the observation that delay tuning in most IC facilitated neurons does not change with increasing sound level (Macias et al., 2012). These features of VNLLc neurons strengthen the conclusion that they provide the critical glycinergic inputs underlying combination-sensitive facilitation in IC (Figure 12).

In other species, VNLL neurons are not segregated so clearly by functional properties or by transmitters, but some VNLL neurons display similar onset response properties (Batra and Fitzpatrick, 1999; Zhang and Kelly, 2006) and may be glycinergic (Saint Marie and Baker, 1990; Saint Marie et al., 1997; Riquelme et al., 2001). Given the topographically complex organization of frequency in this nucleus, it seems reasonable to propose that such neurons could play similar spectral integrative roles in their projections to the IC.

## Processing of combination-sensitive responses beyond the midbrain

The spectro-temporal integrating mechanisms that occur in the auditory brainstem and midbrain appear to explain the basic elements of combination-sensitive responses observed in the auditory thalamus and cortex. Thus, in physiological studies of the mustached bat, auditory midbrain responses show the full range of frequency interactions, the low-frequency inhibition at 0 ms, and the range of best delays of facilitation that have been observed in auditory thalamus or cortex (Portfors and Wenstrup, 2003; Hagemann et al., 2011; Wenstrup and Portfors, 2011; Macias et al., 2012). Anatomical studies in this species show that combination-sensitive regions of the IC project to comparable regions of the medial geniculate body (MGB, Frisina et al., 1989; Wenstrup et al., 1994; Wenstrup and Grose, 1995) and that the combination-sensitive regions in MGB project to the appropriate cortical combination-sensitive areas (Pearson et al., 2007). There is thus strong but indirect support that cortical responses could be inherited from their midbrain and thalamic precursors.

Is there additional processing of combination-sensitive responses beyond the midbrain? The answer appears to be yes for some features of the combination-sensitive response. Thus, there is a greater likelihood, compared to midbrain neurons, that combination-sensitive neurons in MGB will not respond to separate signals, but only respond to the appropriate combination of signals (Yan and Suga, 1996a; Portfors and Wenstrup, 1999, 2003; Wenstrup, 1999). FM–FM neurons in auditory cortex are more likely to respond to FM–FM combinations than to separate elements, to display preferences for FM sweeps rather than to tonal stimuli (Taniguchi et al., 1986; Hagemann et al., 2011; Macias et al., 2012), and to respond to more than one FM harmonic in the echo (Misawa and Suga, 2001). Among cortical FM–FM neurons, delay tuning is more dependent on sound level than in IC (Hagemann et al., 2011; Macias et al., 2012). Finally, cortical FM–FM neurons are more likely to show longer term changes in delay tuning as the result of conditioning or other experience (Yan and Suga, 1996b; Suga et al., 2002; Xiao and Suga, 2004).

The mechanisms underlying these transformations are not understood. A parsimonious hypothesis is that the additional features of cortical responses are layered onto the fundamental response properties established in the brainstem and midbrain. However, auditory cortical neurons receive multiple inputs that may eliminate selectivity apparent in some of the inputs. Based on their work in the pallid bat, Fuzessery and co-workers (Razak and Fuzessery, 2009; Fuzessery et al., 2011) have proposed that selectivity for the rate and direction of FM sweeps, which exists among IC neurons, may be at least partially re-created in the auditory cortex through GABAergic mechanisms. The functional implication of this re-creation is not understood. In the mustached bat, the modifications introduced beyond the auditory midbrain may create response properties that can be modified by experience. Further work is needed to clarify these issues.

## Overall view and implications

Studies in the mustached bat provide an extensive description of the mechanisms of spectro-temporal integration acting in the mammalian auditory brainstem and midbrain. Although the mechanisms of facilitation and delay tuning are not completely understood, it is possible to draw several conclusions regarding auditory brainstem mechanisms underlying spectro-temporal integration. These conclusions point to computational mechanisms operating in the auditory brainstem and midbrain that may be used for other forms of spectro-temporal integration in other species.

### Sequential combinatorial interactions in the mustached bat

**Figure 14:**
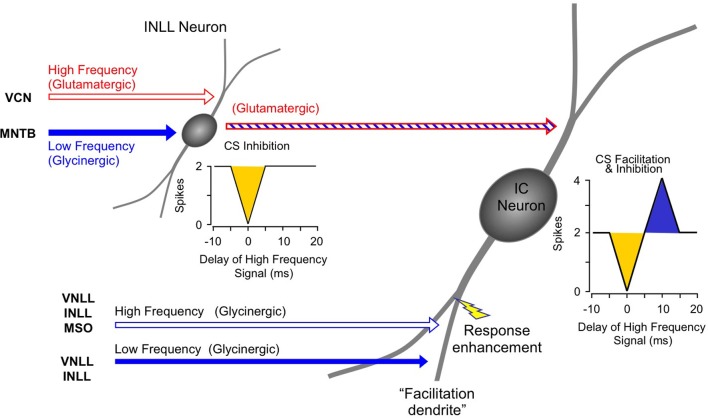
**Schematic diagram of circuitry underlying combination-sensitive facilitation and inhibition in IC neuron**.

The inhibitory and facilitatory interactions that create the response properties observed in IC, MGB, and auditory cortex occur as separate spectro-temporal integrative events within the auditory brainstem and midbrain (Figure 14). Combination-sensitive inhibition is mostly created at a lower level, primarily within the INLL, and depends on integration of high-frequency-tuned excitatory inputs and low-frequency-tuned inhibitory inputs. Combination-sensitive facilitation is created in the auditory midbrain and depends on differently tuned glycinergic inputs. Facilitatory IC neurons appear to receive inputs from different tonotopic regions of the VNLL and INLL (Figure 14). While they also receive other inputs, including glutamatergic inputs, only their glycinergic inputs appear to contribute to combinatorial response properties.

A subset of facilitatory neurons in IC, MGB, and auditory cortex also display inhibitory combination sensitivity. These likely arise in IC as a result of the convergence of a glutamatergic input from inhibited combination-sensitive INLL neurons with the glycinergic inputs from VNLL and INLL that create facilitation. The irony here is that some glycinergic (“inhibitory”) inputs participate in response facilitation while the glutamatergic (“excitatory”) input conveys inhibitory combination-sensitive responses from INLL. This irony is a caution to circuit analyses: sources of “inhibition” and “excitation” to a particular neuron's response should be corroborated by mechanistic studies that establish whether inhibition or excitations are acting at that site.

The interactions that create combination-sensitive responses in the mustached bat appear more hierarchical and the mechanisms appear more unitary than those observed in relation to some other complex response properties observed in IC, such as binaural processing or FM sweep selectivity (Fuzessery et al., 2011; Pollak et al., 2011; Pollak, 2012). One explanation for the well-defined set of interactions underlying combination sensitivity is that the system appears to be prewired before the first experience with echolocation. Thus, tuning to pulse-echo delay occurs in the auditory cortex on the first day after birth, and many features of mature responses are evident within the first week (Vater et al., 2010; Kössl et al., 2012). It is not clear whether this tuning precedes combination sensitivity in the IC, but our hypothesis is that the brainstem and midbrain mechanisms and circuitry form during prenatal development.

### New roles for brainstem nuclei underlying spectral integration

Spectral integration depends on convergence of information from neurons that are tuned to different frequencies. For many neurons in the auditory system, these interactions may result from so-called lateral excitatory or inhibitory effects, in which the inputs are tuned to adjacent or overlapping frequency bands. However, many studies in non-echolocating species show that neurons, particularly in auditory cortex, respond to sounds in distinct frequency bands (Sutter and Schreiner, 1991; Rauschecker et al., 1995; Brosch et al., 1999; Sadagopan and Wang, 2009). These interactions are evident in physiological studies but are difficult to demonstrate anatomically.

As a form of spectral interaction, combination sensitivity in the mustached bat is noteworthy because it involves widely separated frequency bands that may be more amenable to experimental study. The work summarized here shows that most combination-sensitive interactions depend on projections from neurons tuned to a specific lower frequency band (~23–30 kHz) and that these projections occur in specific auditory brainstem nuclei. Thus, for combination-sensitive inhibition, low-frequency neurons in MNTB project to high-ChF neurons in INLL. For combination-sensitive facilitation, low-frequency-tuned neurons in VNLL project onto high ChF neurons in IC. These spectrally unmatched projections in the auditory brainstem have not been reported previously, but may underlie a variety of spectro-temporal integrative responses, from asymmetric inhibitory sidebands (Fuzessery et al., 2011), to facilitation between adjacent frequency bands within an FM sweep (Razak and Fuzessery, 2008), to inhibition that may contribute to other spectral responses (Xie et al., 2007).

The work presented in this review identifies a new role for the MNTB that involves spectro-temporal integration. The MNTB is normally associated with binaural comparisons of similarly tuned inputs (Boudreau and Tsuchitani, 1968; Guinan et al., 1972; Kuwabara and Zook, 1992; Sanes and Friauf, 2000; Thompson and Schofield, 2000; Brand et al., 2002; Kim and Kandler, 2003; Pecka et al., 2008). More recent work shows that MNTB contributes to the offset response of neurons in the superior paraolivary nucleus, a major source of GABAergic inhibition to the IC (Kadner et al., 2006; Kulesza et al., 2007). The work on mustached bats demonstrates that some MNTB neurons project to unmatched frequency representations of target nuclei, creating specific forms of cross-frequency interactions. This is a role that should be explored further in other mammalian species, due to the widespread projections of MNTB to superior olivary and lateral lemniscal nuclei.

This review also describes specific functional roles for neurons in the ventral and intermediate nuclei of the lateral lemniscus. VNLL, especially the columnar subdivision, appears to contribute to combination-sensitive facilitation through convergence of glycinergic low-frequency (23–30 kHz) and high-frequency neurons onto high-ChF neurons in IC. Projections from VNLL that are not matched to the ChF of target IC neurons may underlie the inhibition that contributes to FM sweep selectivity in other bat species (Voytenko and Galazyuk, 2007; Pollak et al., 2011; Williams and Fuzessery, 2011). INLL performs an important spectral integrative function as the initial site where combination-sensitive inhibition arises. Through its excitatory projection, the INLL appears to convey a particular response feature to IC neurons—combination-sensitive inhibition.

### Critical roles of glycinergic neurons in spectral integration

These studies show a predominant role for brainstem glycinergic neurons in the spectral interactions that underlie combination sensitivity. Low frequency tuned glycinergic neurons from MNTB create well timed, predominantly onset inhibition of excitatory responses tuned to frequency bands 1–3 octaves higher. The MNTB low-frequency input provides fast inhibition to suppress the response to a simultaneously delivered high-frequency signal.

The role of glycinergic inputs in combination-sensitive facilitation demonstrated in these studies reveals a novel mechanism of response facilitation/excitation. While it is well known that glycinergic or GABAergic inputs can create excitation or facilitation (Casseday et al., 1994; Wenstrup and Leroy, 2001; Person and Perkel, 2005), the studies reviewed here show that facilitation depends entirely upon glycinergic inputs. We have speculated that these inputs, which appear to result from distinctly tuned glycinergic inputs, create a form of spectral integration that is transient and locked to signal onset. Further, since glycinergic VNLLc neurons respond well at high repetition rates and are phasic (Covey and Casseday, 1991), their synapses onto IC neurons may be more resistant to synaptic fatigue than glutamatergic synapses at the high stimulation rates that can occur during echolocation (Sanchez et al., 2008). The fast action of glycinergic synapses and the phasic release of glycine by VNLL neurons may be optimal for the form of combination sensitivity displayed by these neurons. The glycinergic facilitation mechanism may also underlie the sub millisecond facilitation reported in the pallid bat IC (Razak and Fuzessery, 2008).

For many neurons, delay tuning requires a delayed excitation evoked by the low-frequency input, so that facilitation is strongest when the high-frequency signal occurs several ms later (e.g., resulting from pulse-echo delay). The mechanism underlying the timing of the facilitation is not understood, but our data suggest it does not depend on glutamatergic or GABAergic inputs to the IC facilitated neurons. We have speculated that the glycinergic rebound excitation may follow a variable period of increased chloride conductance that creates the opportunity for delayed low-frequency excitation. Further research is needed here, but it has the potential to reveal interesting postsynaptic mechanisms of information processing within IC neurons.

### Segregated signal processing by IC neurons

The nature of facilitatory spectral integration by IC neurons in the mustached bat has led us to propose that individual neurons perform different signal processing operations in different cellular compartments, or processing domains (Figure 14; Peterson et al., 2008; Sanchez et al., 2008). Specifically, we propose that inputs onto IC neurons are anatomically and electrically isolated to preclude immediate interactions with other inputs. These different sites integrate information from different sets of inputs, and may operate under different conditions. For example, during roosting, bats primarily hear social vocalizations. These generally have lower repetition rates than those during echolocation in pursuit of insects. IC neurons would likely respond to these social signals through activation of their ChF-tuned glutamatergic, GABAergic, and glycinergic inputs. These inputs would also be influenced by frequencies within the very low-frequency (<23 kHz) tails of the high-ChF tuning curves, which activate excitation and/or suppression (Marsh et al., 2006; Nataraj and Wenstrup, 2006). During sonar behavior, stimulation rates that exceed 40/s may result in reduced responsiveness to the glutamatergic inputs. Under these conditions, the same neuron may be activated primarily by its glycinergic inputs related to combination sensitivity, and the delay tuning resulting from the glycinergic inputs would dictate the overall response. Our data suggest that these sets of inputs do not appear to interact, leading to largely independent processing of acoustic stimuli in a context-dependent fashion.

### Conflict of interest statement

The authors declare that the research was conducted in the absence of any commercial or financial relationships that could be construed as a potential conflict of interest.
